# Effects of maternal sugar intake during pregnancy on allergies in offspring: A protocol for systematic review and meta-analysis

**DOI:** 10.1097/MD.0000000000027447

**Published:** 2021-10-22

**Authors:** Yijun Wu, Xiaoli Chai

**Affiliations:** a Department of Obstetrics, Huanggang Central Hospital, Huanggang, Hubei, China; b Department of Obstetrics and Gynecology, Xishui County People's Hospital, Hubei University of Science and Technology, Huanggang, Hubei, China.

**Keywords:** allergy, meta-analysis, pregnancy, protocol, sugar intake

## Abstract

**Background:**

To our knowledge, there is no comprehensive evidence quantifying the plausibility of the association between maternal free sugar intake during pregnancy and the development of offspring's allergic diseases. Thus, this systematic review examines the weight of evidence for causality from cohort studies on the association between maternal free sugar intake during pregnancy and the development of allergies in offspring.

**Methods:**

The present meta-analysis is prepared and reported in accordance with the Preferred Reporting Items for Systematic Reviews and Meta-analyses guidelines. We search PUBMED, Scopus, EMBASE, and Cochrane Library databases through October, 2021. All clinical trials to assess the efficacy of maternal sugar intake during pregnancy on allergies in offspring are considered eligible for analysis. The Comprehensive Meta-Analysis Software 2 program is used for statistical analyses of the pooled data. A *P* value < .05 is considered statistically significant. The outcome measure is development of allergic disease among offspring assessed by any method (parental history, doctor diagnosed, or questionnaire based).

**Results:**

It is hypothesized that high free sugar consumption during pregnancy may be associated with the development of allergies in offspring.

## 1 Introduction

Our food supply has been largely processed into higher levels of sugar than in previous generations. Numerous studies have examined the link between excessive sugar consumption and the risk of obesity and a host of other health problems.^[1–3]^ This has included consideration of both regular dietary sugars (sucrose, fructose, etc) and alternative sweeteners, encompassing artificial sweeteners (aspartame, sucralose, etc) and natural low or zero energy sweeteners (stevia, sugar alcohols, etc), that are abundant in the food supply. However, the concept of sugars and alternative sweeteners as environmental exposures that can have secondhand effects on the developing fetus has not been widely explored.^[4]^

There is considerable interest in the role of maternal diet in the etiology of allergy in offspring. From 1970 to 2000, per capita consumption of all refined sugars in the United States increased by 25%, matching global trends.^[5]^ Current international dietary guidelines advise people to reduce their consumption of sugar, and more particularly free sugars, which comprise sugars (monosaccharides and disaccharides) added to foods or drinks by the manufacturer, cook or consumer, and sugars naturally present in honey, syrups and unsweetened fruit juices.^[6,7]^ Results from previous studies suggested there might be a critical window during pregnancy when consuming foods and nutrients, including sugar, puts a developing fetus at risk for allergies.^[8–11]^

To our knowledge, there is no comprehensive evidence quantifying the plausibility of the association between maternal free sugar intake during pregnancy and the development of offspring's allergic diseases. Thus, this systematic review examines the weight of evidence for causality from cohort studies on the association between maternal free sugar intake during pregnancy and the development of allergies in offspring.

## 2 Materials and methods

### 2.1 Study registration

The present meta-analysis is prepared and reported in accordance with the Preferred Reporting Items for Systematic Reviews and Meta-analyses guidelines. The research question is defined by the participants, interventions, comparisons, outcomes, and study (PICOS) design. Two investigators independently perform the literature search, study selection, data extraction, and quality assessment. Discrepancies between the investigators are resolved by mutual consent. The systematic review protocol has been registered on Open Science Framework registries. Ethical approval and patient consent are not required because this study is a literature-based study. We will update our protocol for any changes in the entire research process if needed.

### 2.2 Data sources and search strategy

We search PUBMED, Scopus, EMBASE, and Cochrane Library databases through October 2021. Search algorithm are identified as follows: (sugar) OR (sweet) OR (fructose) AND (pregnancy) OR (matern) OR (antenatal) AND (asthma) OR (eczema) OR (allergy). The literature search, data extraction, and quality assessments are conducted independently by 2 authors. We also search references cited in all included articles to avoid missing other relevant articles. If the effective data are not included in the original articles, we will contact the authors to get them. The studies are screened and evaluated by 2 authors independently for eligibility.

### 2.3 Eligibility criteria

Study included in our meta-analysis have to meet all of the following inclusion criteria: all clinical trials to assess the efficacy of maternal sugar intake during pregnancy on allergies in offspring are considered eligible for analysis; pregnant mothers and offspring (median age from 0–5 years of age); the outcome measure is development of allergic disease among offspring assessed by any method (parental history, doctor diagnosed, or questionnaire based). Studies with overlapping data or insufficient data to calculate or extract effect estimates are excluded. Case reports, biochemical trials, letters, and reviews are also eliminated.

### 2.4 Data extraction

After discarding the duplicate studies, 2 reviewers independently evaluate the potentially eligible studies. The studies are screened for eligibility based on a review of the title and abstract, and disagreements are resolved through consensus. After screening, 2 independent reviewers read the full texts of the studies and re-assessed the eligibility of each. Subsequently, the data, including name of the first author, publication date, study type, number of patients, demographic information (age and sex), follow-up duration, adjusted factors, and outcome data, are extracted. The outcome measure is development of allergic disease among offspring assessed by any method (parental history, doctor diagnosed, or questionnaire based).

### 2.5 Statistical analysis

The Comprehensive Meta-Analysis Software 2 program is used for statistical analyses of the pooled data. In each analysis, a heterogeneity test is performed using I^2^ statistics, which measures the extent of inconsistency among the results. I^2^ values > 50% are considered to indicate substantial heterogeneity and the random-effects model is used for analysis of the data. In contrast, when the I^2^ value is < 50%, the pooled data are considered homogeneous, and a fixed-effect model is applied. Additionally, 95% CIs are used in the analysis. A *P* value < .05 is considered statistically significant. Funnel plots are used to evaluate possible publication bias.

### 2.6 Quality assessment

In order to achieve a consistency (at least 80%) of risk of bias assessment, the risk of bias assessors will pre-assess a sample of eligible studies. Results of the pilot risk of bias will be discussed among review authors and assessors. Two independent reviewers will assess the risk of bias of the included studies at study level. We will follow the guidance in the latest version of Cochrane Handbook for systematic reviews of interventions when choosing and using tools to assessing risk of bias for randomized trials (version 2 of the Cochrane risk of bias tool for randomized trials, RoB 2) and non-randomized trials (the Risk Of Bias In Non-randomized Studies of Interventions, ROBINS-I tool). Any disagreements will be discussed and resolved in discussion with a third reviewer. Studies with high risk of bias or unclear bias will be given less weight in our data synthesis.

## 3 Discussion

Several theories suggest that sugar consumption has a mechanistic role in the development of allergic diseases. Sugar is said to activate inflammation throughout the body or trigger symptoms that lead to allergies.^[12]^ Another potential mechanism is the possible triggering of allergic reactions through the mediating role of overweight and obesity.^[13]^ There is substantial evidence showing that drinking sugary beverages can lead to overweight and obesity in children and adults.^[14,15]^ It has been speculated that a mother's diet during pregnancy may regulate the immune system development of her offspring and contribute to the development of allergic diseases in offspring through overweight and obesity.^[16]^ However, the evidence for these mechanisms remains inconclusive.

## Author contributions

**Conceptualization:** Xiaoli Chai.

**Data curation:** Yijun Wu.

**Formal analysis:** Yijun Wu.

**Funding acquisition:** Xiaoli Chai.

**Investigation:** Yijun Wu.

**Methodology:** Xiaoli Chai.

**Project administration:** Xiaoli Chai.

**Writing – original draft:** Yijun Wu.

**Writing – review & editing:** Xiaoli Chai.
